# COMPARING ILLNESS PERCEPTIONS OF MULTIMORBIDITY AMONG OLDER ADULT UTILIZERS AND NON-UTILIZERS OF HOME-BASED CARE

**DOI:** 10.1093/geroni/igad104.3238

**Published:** 2023-12-21

**Authors:** Ayomide Bankole

**Affiliations:** UNC Chapel Hill, Chapel Hill, North Carolina, United States

## Abstract

Health behavior interventions targeting illness perceptions improve outcomes for those with chronic illness. Research investigating illness perception in the context of multimorbidity suggests illness-perception of multimorbidity (IP-MM) predicts short-term health status among individuals with multimorbidity. However, less is known about IP-MM among home-based care (HBC) utilizers who receive in-home support for multimorbidity management. This study aimed to compare IP-MM among older adults HBC-utilizers and non-utilizers. Secondary analysis of a cross-sectional study exploring IP-MM in 116 community-dwelling older adults with ≥2 chronic conditions. IP-MM was assessed via MULTIPleS summary and subscale scores (0-78), including causal-links (0-9), prioritization (0-12), treatment-burden (0-18), activity-restrictions (0-9), and emotional-representations (0-30), where higher scores indicate stronger perceptions. HBC-utilizers self-reported receiving nursing, rehabilitative, or medical services at home. One sample t-tests was performed to compare MULTIPLeS subscales between HBC-utilizers and non-utilizers. HBC utilizers comprised of 18% with 82% as non-utilizers. Mean number of chronic conditions was 4.5 (2); Mean age was 73.3 (6.72), 71% female, 35% Hispanic, 33% Black, 27% White, and 5% Asian. Mean (SD) MULTIPleS summary score was 39.39 (21.1). Subscales scores:[activity restriction: 5.19 (2.88); causal relationships: 4.81 (3.07); prioritization:8 .24 (3.52); treatment burden: 7.84 (5.57). HBC utilizers had significantly higher mean treatment-burden subscale scores (10.38 ± 5.51), than non-utilizers (7.28 ± 4.45), p=0.027. No significant differences in other subscales or summary scores. Older adult HBC-utilizers and non-utilizers experienced comparable IP-MM, differing only in treatment burden perceptions. Additional research on IP-MM is needed to develop interventions that improve multimorbidity management and treatment-burden among HBC utilizers.

